# The Effects of Hydration Status on Cognitive Performances among Young Adults in Hebei, China: A Randomized Controlled Trial (RCT)

**DOI:** 10.3390/ijerph15071477

**Published:** 2018-07-12

**Authors:** Jianfen Zhang, Na Zhang, Songming Du, Hairong He, Yifan Xu, Hao Cai, Xiaohui Guo, Guansheng Ma

**Affiliations:** 1 Department of Nutrition and Food Hygiene, School of Public Health, Peking University, 38 Xue Yuan Road, Haidian District, Beijing 100191, China; ZJF@bjmu.edu.cn (J.Z.); ziqingxuanping@126.com (N.Z.); hhrhhr3@163.com (H.H.); xuyifan_1992@163.com (Y.X.); caihao169@pku.edu.cn (H.C.); guo-xiaohui@163.com (X.G.); 2 Laboratory of Toxicological Research and Risk Assessment for Food Safety, Peking University, 38 Xue Yuan Road, Haidian District, Beijing 100191, China; 3 Chinese Nutrition Society, 6 Guang An Men Nei Street, Xicheng District, Beijing 100053, China; dusm9709@126.com

**Keywords:** water, dehydration, water supplementation, cognitive performances, magnetic resonance imaging

## Abstract

*Background*: Dehydration may affect cognitive performances as water accounts for 75% of brain mass. The purpose of this study is to investigate the effects of dehydration and water supplementation on cognitive performances, and to explore the changes of brain structures and functions using MRI. *Methods and Analysis*: A double-blinded randomized controlled trial has been designed and will be implemented among 64 college students aged 18–23 years from Baoding, China. Subjects will be asked to restrict water for 36 h. The first morning urine will be collected and urine osmolality measured. The fasting blood samples will be collected and osmolality and copeptin will be measured. Three MRI sequences, including fMRI, ASL and 3D BRAVO will be taken to observe the changes of whole brain volume, ventricular volume, BOLD response and the cortex thickness. Cognitive performances and mood will be performed with software and questionnaires, respectively. Subjects in the water supplementation groups 1, 2, 3 will drink 200, 500 and 1000 mL of water, respectively, while subjects in the no water supplementation group will not drink any water. After 90 min, urine and blood samples, MRI scans, cognitive performances and mood will be performed. One-way ANOVA will be used to study the differences among groups. *Ethics and Dissemination*: The study protocol has been approved by the Peking University Institutional Review Committee. Ethical approval project identification code is IRB00001052-16071. Results will be published according to the CONSORT statement and will be reported in peer-reviewed journals.

## 1. Introduction

Water, which makes up about 60–70% of human body weight [1], participates in metabolism, maintains electrolyte balance, and has other functions. It is very important for humans to maintain the balance of water input and output. When the water input is less than the output, people may be dehydrated. Dehydration may lead to some adverse results such as decreased muscle endurance and strength [2], or increased chances of kidney stones and urinary tract infections [3]. Furthermore, dehydration may affect cognitive performances, as water accounts for 75% of brain mass [4].

A few investigators have studied the effects of dehydration on human cognitive performances. However, there have been no consistent results and conclusions. Some studies have shown that dehydration impaired the cognitive performances in the aspects of short-term memory [5], vigilance attention, choice reaction [6], or working memory [7]. Other studies presented contradictory findings [8,9]. Mild dehydration affected the visual vigilance and visual working memory response latency of men [7], but without substantially altering the key aspects of cognitive performances of women [10], which indicated that there exist differences between genders that need to be studied further. The studies mentioned above used heat-stress, intense exercise, diuretics, or fluid deprivation or combined them to induce a dehydrated status. However, heat stress [11] or exercise [12] could also affect cognitive performances, which makes it difficult to investigate the effect of dehydration alone. When referring to the effects of hydration induced by fluid restriction alone, some studies found changes of moods [13,14,15], but the cognitive performances were not affected. More studies are needed to investigate the effects of dehydration induced by fluids restriction on cognitive performances.

Several studies have investigated the effects of hydration on brain volume and other changes of brain, which also gave conflicting results. Duning et al. [16] analyzed three-dimensional data sets at three time points: (1) before (baseline) and (2) after water restriction for 16 h, and (3) 20 to 30 min after drinking (rehydration). They found that after water restriction for 16 h, the brain volume had a decrease of 0.55% when compared with the baseline, moreover, after rehydration, the volume of brain increased 0.72% when compared with the baseline. Kempton et al. [17] used 90-min physical exercise to induce dehydration, and the results showed that acute dehydration could lead to the ventricular expansion, but there was no change in the volume of the total brain, which was different with Duning’s study. Nakamura et al. [18] found that the brain volume significantly increased by about 0.36% between the dehydrated and rehydrated states, but did not change significantly during the dehydration interval. The research, which was conducted among 10 adolescents (half males and half females), demonstrated that the dehydration, induced by thermal exercise, led to a significantly stronger increase in blood-oxygen-level-dependent (BOLD) responses during an executive function task than the control condition [19]. In a study conducted among 15 healthy individuals, the results showed that the brain tissue fluid had an decrease of 1.63% after fluids restriction for 12 h [20]. The conclusions of the above-mentioned studies were different presumably due to the confounding factors such as the different methods to induce dehydration, the different volumes of water intake, and the different intervals after water supplementation, which needs more studies with appropriate study designs.

Studies about the effects of dehydration on cognitive performances and mood have not been much reported. In the status of dehydration, the changes of whole brain volume, ventricular volume, BOLD response, the thickness of the cortex observed using Magnetic Resonance Imaging (MRI), have not been explored in China. In addition, the effects of different volumes of water supplementation after dehydration on cognitive performances and mood have not been investigated in China.

Referring to the effects of dehydration on cognitive performances, there was only one study conducted in China, which was designed and implemented by our team. It was a randomized controlled trial conducted among 68 college students. The results showed that water supplementation of 400 mL could improve the adverse effects of hypohydration on cognitive performances (unpublished). However, the study only investigated males. The level of dehydration conducted before was mild, however, and moderate dehydration was not investigated. Moreover, the changes of brain structures and functions in dehydration and water supplementation by MRI have not been explored yet. Hence, we designed a randomized controlled trial to investigate the effects of dehydration after 36 h of water restriction and rehydration after water supplementation on cognitive performances and the changes of brain structures and functions in different hydration status among male and females in free-living conditions.

We hypothesize that dehydration has adverse effects on cognitive performances and mood. Dehydration will induce the decrease of whole brain volume and the increase of ventricular volume, the BOLD and the cortex thickness. After water supplementation, the cognitive performances and mood will be improved. There will be an increase in the whole brain volume and decreases in ventricular volume, BOLD, and the cortex thickness. Moreover, there will be significant differences in the effects of dehydration on aspects of cognitive performances, mood, and the structures and functions of the brain between males and females in dehydration and water supplementation.

The purposes of this study are, first, to evaluate the effects of dehydration induced by 36 h water deprivation on cognitive performances and mood. Second, to explore whether cognitive performances and mood will be improved after water supplementation. Third, to compare the changes of whole brain volume, ventricular volume, BOLD response and the thickness of the cortex using MRI between dehydration and rehydration. Fourth, to compare the differences between males and females. Finally, to provide more suggestions for the associations between water intake and cognitive performances, may offer more scientific basis to revise the adequate water intake for young adults.

## 2. Methods and Analysis

### 2.1. Sample Selected

Subjects will be recruited using both online and campus recruitment advertisements among young adults from Hebei University Health Science Center, which is located in Baoding, China. All subjects will sign the informed consent form before participating the study. Anyone aged <18 years or >23 years or smoke, has habitual alcohol (>20 g/day) consumption or habitual high caffeine (>250 mg/day) consumption or intensive physical exercise, or has a chronic diseases or claustrophobia or any other neurologic, medical illness will be excluded from participation. Subjects will be instructed not to participate in strenuous activities, to maintain a normal diet and to avoid alcohol consumption during the 24 h before.

### 2.2. Sample Size Calculation

In a related study, the letter cancellation scores of subjects in the water group before and after water supplementation were 23.34 and 28.81, respectively [21]. According to the SAS procedures (SAS Institute Inc., Cary, NC, USA), with the power set at 0.8 and α set at 0.05, in addition, a 10% drop-out rate will be taken into account, so a total of 64 subjects will be needed, including half males and half females.

### 2.3. Ethics and Dissemination

All subjects will give their informed consent for inclusion before they participate in the study. The study will be conducted in accordance with the Declaration of Helsinki, and the protocol has been approved by the Peking University Institutional Review Committee. Ethical approval project identification code is IRB00001052-16071.

### 2.4. Study Design

**Figure 1:**
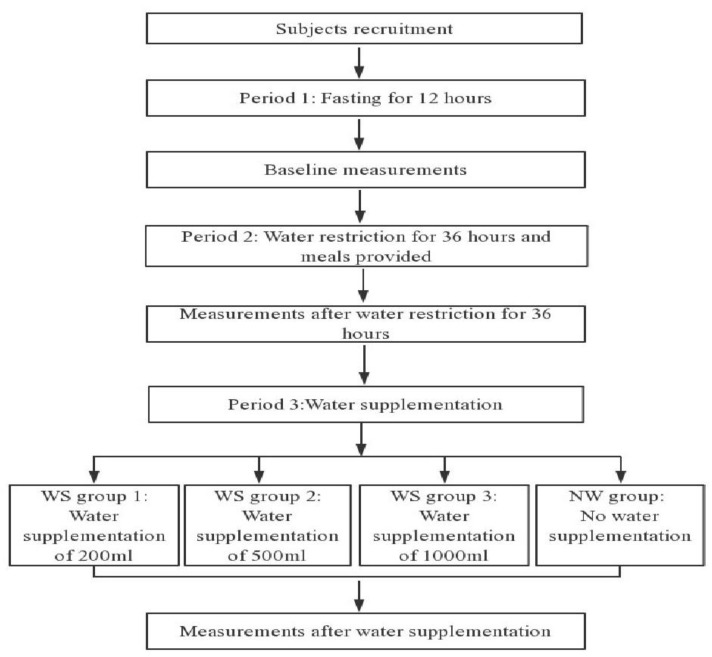
Study design.

A randomized controlled trial study design, which is double-blinded, will be employed. All recruited subjects will fast overnight for 12 h, without consuming any food or drink. They will be asked to have the meals provided by the study. Subjects will be randomly assigned into four groups using a random number generated by computer software: the water supplementation group 1, 2, 3 (WS group 1, 2, 3) with 200 mL (WS group 1), 500 mL (WS group 2) and 1000 mL (WS group 3) water, respectively and the no water supplementation group (NW group). The present study will assess cognitive performances, mood, subjective sensation of satiety, hunger, sleepiness and thirst during the experimental design three times. Moreover, MRI scans will be used to explore the changes of brain structures and functions for three times. In addition, measurements of weight, waist circumference, body composition, blood pressure, blood glucose, urine osmolality, urine specific gravity, plasma osmolality, and plasma copeptin will be executed at the same time. The study design is shown in Figure 1. All items from the World Health Organization Trial Registration Data Set are shown in Table 1.

### 2.5. Randomization and Blinding

This will be a randomized double-blinded study, and subjects will be randomly assigned into different groups using a random number generated by the computer software. The assignment will be stored in numbered, sealed, opaque envelopes, which will be prepared by an investigator, who is not involved in assessments and subject recruitment. The envelopes will be opened by the investigator. The investigators who will complete the statistical analysis will be blinded to the data collection and data entry, with each subject identified by a random number. Moreover, when taking water supplementation, every subject will be given three opaque cups. The water supplied to the subjects in water supplementation will be contained in the three opaque cups, and subjects will be told to drink the water from the little hole on the cover of the cup within 10 min. After water supplementation, they are not allowed to talk with each other about the interventions they have had.

### 2.6. Study Procedure

The study will be consisted of three periods:

Period 1: from 8:00 p.m. of the first study day to 8:00 a.m. of the second study day. Subjects will be asked to fast overnight for 12 h, without consuming foods or drinks. Before 8:00 p.m. of the first study day, subjects are instructed to having fluid intakes as before. Strenuous exercise will not allowed in the 24 h before the study. The subjects will be asked to sleep before 11:00 p.m. and are not allowed to urinate before they get up in the morning. Anyone who fails to meet the above requirements has to let the investigators know.

Period 2: from 8:00 a.m. of the second study day to 8:00 a.m. of the third study day. Subjects will be asked to have water restriction for 24 h. Meals containing ≤75% of water will be provided by the study. All foods will be weighted before and after the subjects eat, with the aim of assessing the intake of water from food of the subjects. Baseline measurements including height, weight, waist circumferences, body composition, blood pressure, blood glucose and blood samples will be obtained following standardized procedures. The first urine samples will be collected and tested. Subjects will have MRI scans for about 15–20 min. All subjects will have two medical earplugs and will not be allowed to sleep or move, just lying down for 15–20 min. Tests of mood, cognitive performances and subjective sensation will be performed by investigators. During Period 2, the urine samples will be collected and sent to the hospital laboratory immediately to measure the related indexes. They will also be asked to go to bed before 11:00 p.m. and are not allowed to urinate before they get up in the morning.

**Figure 2:**
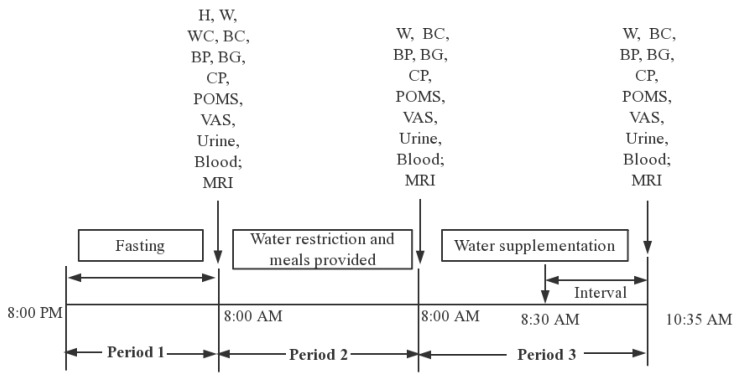
The study procedure (Note: H (Height); W (Weight); WC (Waist circumference); BP (Blood pressure); BG (Blood glucose); BC (Body composition); CP (Cognitive performances); POMS (Profile of Mood States); VAS (Visual Analogue Scales); MRI (Magnetic resonance imaging)).

Period 3: from 8:00 a.m. of the third study day to 10:35 a.m. of the third study day. Subjects will have water supplementation. They will be asked to arrive at the lab at 8:00 a.m. Measurements including weight, body composition, blood pressure, blood glucose and blood samples will be obtained as before. The first morning urine and blood samples will also be collected and tested. MRI scans, cognitive performances, mood and subjective sensation of the subjects will be performed. At 8:30 a.m., subjects in WS group 1, WS group 2, WS group 3 will be asked to drink 200, 500, and 1000 mL of purified water, respectively. The subjects in NW group will drink no water or other fluids. All the water will be offered in opaque containers which should be drunk within 10 min. All the water will be kept in 30 °C~40 °C to reduce the gastrointestinal discomfort of the subjects. During the interval of 90 min, all subjects will be asked to undertake normal activities in the room adjacent to the testing room. After water supplementation for 90 min (at 10:15 a.m.), weight, body composition, blood pressure and blood glucose will be measured. Urine and blood samples will be collected, stored and tested. MRI scans, cognitive performances, mood and subjective sensation will be measured again. The study procedure is shown in Figure 2.

Subjects will be asked everyday if they have fever, diarrhea or other illness during the study. During the three periods of the study, all subjects will not be allowed to undertake strenuous activities. After water supplementation, the gastrointestinal distress questionnaire will be conducted among the subjects to evaluate the discomfort.

The schedule of enrolment, interventions, and assessments is shown in Table 2.

### 2.7. Anthropometric Measurements

Height (H), waist circumference (WC), blood pressure (BP) (systolic pressure: SP; diastolic pressure: DP) and blood glucose (BG) will be measured by trained investigators using professional instruments, respectively, as showed in our previous study [22] (HDM-300; Huaju, Zhejiang, China; MyoTape waistline measurer; HEM-7051; Omrom, Jiangsu, China; One Touch Ultra Easy; Johnson, Shenzhen, China). (BMI: weight (kg)/height squared (m)); Body surface area (m^2^): (male: weight (kg)×0.0021+height (cm)×0.0057+0.0882, female: weight (kg)×0.0127+height (cm)×0.0073−0.2106) [23]. Body composition (BC) will be measured by trained investigators using human body composition analyzer (Inbody 720, Biospace, Seoul, Korea).

### 2.8. Urine and Blood Biomarkers

Urine will be collected by the subjects using self-designed containers and sent to the laboratory and be tested immediately. All urine samples will be stored at +4 °C before measurements. Urine osmolality will be assessed with the freezing point method by osmotic pressure molar concentration meter (SMC 30C; Tianhe, Tianjin, China). Urine specific gravity (USG) and urine pH will be tested by automatic urinary sediment analyzer with uric dry-chemistry method (H-800; Dirui, Changchun, China). Urine color (UC) will be measured by investigators using chromatogram spectrophotometer (CM-5, Konica Minolta, Tokyo, Japan).

Urine and blood electrolyte concentrations (including sodium, potassium, chloride, calcium, magnesium, and phosphate) will be tested by automatic biochemical analyzer with the ion-selective electrode potentiometer method (AU 5800; Beckman, Brea, CA, USA). Blood urea nitrogen concentration (BUN) and creatinine concentration (Cre) will be measured by automatic biochemical analyzer (AU 5800; Beckman, Brea, CA, USA). Plasma copeptin concentration will be measured using enzyme-linked immunosorbent assay for antigen detection (CEA365Hu, Cloud-Clone, Wuhan, China).

### 2.9. Definition of Hydrated Status

The balance between water output and water input defines the hydration status. Dehydration occurs when the water input is insufficient to replace the free water output, which is defined as the urine osmolality is greater than 800 mOsm/kg [24]. Optimal hydration is defined as the 24-h urine osmolality ≤500 mOsm/kg, middle hydration is defined when 24-h urine osmolality is between 500 mOsm/kg and 800 mOsm/kg [25].

### 2.10. Visual Analogue Scales (VAS) for Subjective Sensation

Subjects will be instructed to place a pencil mark anywhere on the 10-cm-line designed to quantitatively measure the subjective sensation of satiety, hunger [26], sleepiness [27] and thirst [28]. The higher the score, the more hunger, thirsty, and sleepy the subjects are, as shown in our previous study [22].

### 2.11. Profile of Mood States (POMS)

The POMS is a widely used, brief, standard inventory of mood states, including seven subscales on a 5-point, 40 adjectives, which examines tension, depression, anger, vigor and confusion, etc. [29], as shown in our previous study [22].

### 2.12. Cognitive Performances (CP)

Six different cognitive tests are administrated using a “primary cognitive ability” software from the Institute of Psychology, Chinese Academy Sciences (Beijing, China), to investigate the cognitive performances of subjects.

### 2.13. Vocabulary Test

The vocabulary test will be used to assess verbal comprehension. Subjects will be asked to choose the most accurate explanation of a single word displayed on the screen from five options. The test has 36 words, and there is only one most accurate option, two accurate options and two wrong options. When subjects choose the most accurate, accurate and the wrong option, they will receive scores of 2, 1 and 0, respectively [30]. The higher scores they receive, the better ability of verbal comprehension they have.

### 2.14. Similarities Test

Similarities test will be used to assess the verbal comprehension. Subjects will be asked to find the similarities of the two words on the screen and to choose the most accurate explanation of the similarities from five options [30]. The test has 32 vocabularies, and there is only one most accurate option, two accurate options and two wrong options. When subjects choose the most accurate, accurate and the wrong option, they will obtain scores of 2, 1 and 0, respectively. The higher they scores they get, the better ability of verbal comprehension they have.

### 2.15. Symbol Search Test

Symbol search test will be used to measure processing speed during the three periods. There is a line in the middle of the screen with two symbols above the line, and five symbols below the line. Subjects will be asked to find the corresponding symbol between the symbols above and below the line as much as possible within the time (120 s). The higher the scores, the better the processing speed the subjects have [30].

### 2.16. Operation Span Test

Operation span test will be used to evaluate the ability of working memory during the three periods. It consists of mental arithmetic task and remembering the Chinese-zodiac-signs task. Subjects will be asked to do the mental arithmetic task and simultaneously, remember the Chinese-zodiac-signs after each mental arithmetic task, then, subjects will be asked to recall the order of the Chinese-zodiac-signs. Every mental arithmetic task has a time limitation. When the order of the Chinese-zodiac-signs is right, the subjects will obtain 1 mark. The scores of the test is consisted of two parts, only when the correct rate reaches 80% of the total, will the scores be considered as valid scores [31].

### 2.17. Paper Folding Test

Paper folding test will be performed to assess the visual-spatial ability during the three periods. Subjects will be asked to imagine that there is a square paper folded in some way in their mind, and use a pencil to punch a hole in the paper, then open the paper, to imagine how many holes are on the paper. Subjects will choose one from five options. The test has 12 sections, and the order of the test is from easy to difficult. The numbers of the right potions will be calculated as the scores of the subjects, where a higher score is the better [32].

### 2.18. Portrait Memory Test

Portrait memory test will evaluate the ability of episodic memory during the three periods. The test consists of six portraits [33]. The six portraits will be shown one by one on the screen, and the last name, the work and the hobby of each portrait will be shown at the same time. When the six portraits have been shown for once, then, the portrait will occur in a different order. The subjects will be asked to recall the last name, the work and the hobby of the portrait on the screen. Then, the six portraits will showed for the second time in a different order, where the subjects will be asked to recall the last name, the work and the hobby of the portrait on the screen for a second time. When subjects choose the right last name, the right work and hobby of the portrait, they will score 2, 1, and 1, respectively.

### 2.19. The Magnetic Resonance Imaging of Brain (MRI)

All procedures will be handled by experienced technicians. The subjects will be asked not to move or to sleep, and to just lie down on the scanner bed until all the sequences have finished.

MRI is commonly used in studies of neurodegenerative diseases. In order to detect whether the hydration statuses will affect the brain functions, structures and blood flow, three MRI sequences will be used in this study.

Oblique fMRI can measure the neural activity during hydration status. ASL can assess the resting regional cerebral blood flow, and examine observed changes in BOLD signal, which may be due to changes in blood flow. The Oblique fMRI, ASL and A× 3D BRAVO can evaluate the effects of hydration statuses on whole brain volume, ventricular volume, BOLD response and the thickness of the cortex.

Brain image data will be obtained on a 3.0-T system (Discovery MR750 3.0T, GE, Fairfield, CT, USA, 32-channel head-coil) with a T1-weighted sequence by technicians, in the Affiliated Hospital of Hebei University in Baoding, Hebei Province, China. The MRI scans will be made of three sequences: Oblique fMRI (functional magnetic resonance imaging), arterial spin labelling (ASL, non-contrast) and A× 3D BRAVO (axial brain volume).

### 2.20. The Parameters of the Three Sequences

A× 3D BRAVO

The parameters are: Flip angle = 12°, FOV = 25.6 cm, phase FOV = 1.0 cm, slice thickness = 1.0 mm.

3D ASL MRI (Non-contrast, arterial spin labelling)

ASL data will be collected at rest on the same scanner using a pseudo-continuous ASL tagging scheme with a 3D interleaved spiral FSE readout. The parameters are: FOV = 24 cm, slice thickness = 4.0 mm, slice = 36.

Oblique fMRI (functional magnetic resonance imaging)

Oblique fMRI parameters are: TR = 2000 ms, TE = 30 ms, flip angle = 90°, FOV = 24 cm, phase FOV = 1.0 cm, slice thickness = 4.0 mm, spacing = 0, slice = 38.

### 2.21. Temperature and Humidity of the Environment

The temperature and humidity both indoors and outdoors at the study site will be measured using a temperature hygrometer each day during the three periods (WSB-1-H2, Exasace, Zhengzhou, China).

### 2.22. Statistical Analyses

Statistical analyses will be conducted using SAS 9.2 (SAS Institute Inc., Cary, NC, USA). One-way ANOVA or Kruskal-Wallis *H* test measurements will be used to investigate the effect of hydration statuses on the changes in urine indexes, plasma indexes, mood, cognitive performances and MRI scans among the four groups. The MRI data will be analyzed by professional analyst using MATLAB (Mathworks Inc., Natick, MA, USA) and SIENA (FMRIB Software Library, Oxford, UK). Two-sided significance levels will be set at 0.05 (*p* < 0.05) with 95% confidence intervals (95% CI).

## 3. Trial Registration

Chinese clinical trial registry registration number: ChiCTR-IOR-17011568. Date of registration: 15 June 2017.

## 4. Discussion

Hydration is of vital importance for the human body, especially for brain functions. Hence, while a few studies have explored the effects of dehydration on cognitive performance, there have been no consistent findings owing to some confounding factors. Moreover, studies about changes of brain volume and other aspects of the brain were few, which is in need of being more investigated.

In our study, a randomized controlled trial will be conducted to study the effects of dehydration and water supplementation on cognitive performances. The changes of whole brain volume, ventricular volume, BOLD response and the thickness of the cortex between dehydration and water supplementation will be explored.

One challenge we are going to face is the compliance of the subjects and the quality control. In this study, subjects will need to participate and finish at least three periods. Moreover, they will have water restriction for 36 h. Another challenge is that, subjects will be asked not to move, or to sleep when they have MRI scans, which needs them to lie down for 15–20 min. Investigators will fully explain the principles and requirements to the subjects before the study begins to ensure the compliance. Investigators will be in contact with each subject every day. If subject do quit, the investigators will handle it timely and record the time and reasons. The reliability and validity of the cognitive performances software in this study are guaranteed as for the software has been used in many studies among different subjects with different characteristics. The process in this study will be conducted by professional investigators under standard procedure. All staff in the laboratory are experienced to handle all the measurements.

The present study has both strengths and weaknesses. First, a randomized controlled trial, which also is a blind designed study, will be conducted to reduce the bias, such as reporting bias, exposure suspicion bias and diagnostic suspicion bias. The investigators who conduct the measurements and the data analysts will be blinded to the intervention assignments. Secondly, the urine specific gravity, osmolality, and plasma osmolality will provide sufficient power to confirm the hydration statuses of the subjects. Blood urea nitrogen and creatinine will be measured during the dehydration in order to monitor the renal functions of the subjects. Third, the sample size will provide sufficient power to detect the effects. Finally, differences between genders will be evaluated.

In terms of the weaknesses, the effects of long-term water supplementation on cognitive performances will not be studied; more aspects of brain changes will not be explored; and the effects of different temperatures of water will not be investigated.

## 5. Conclusions

In summary, this is the first time that the effects of dehydration induced by long-term water restriction on cognitive performances, and the improvement of cognitive performances after water supplementation will be explored. Moreover, the changes of whole brain volume, ventricular volume, BOLD response and the thickness of the cortex will be observed by first using MRI among young adults in China. The study will provide more profiles for the importance and associations of fluids intake for hydration status and the cognitive performances, may offer more scientific basis to revise the adequate water intake values for Chinese adults and supply more credible theoretical basis for water health education.

## Figures and Tables

**Table 1:** All items from the World Health Organization Trial Registration Data Set (SPIRIT checklist, item 2b).

Data Category	Information
Registration number	ChiCTR-IOR-17011568 Chinese clinical trial registry
Registration State	1008001 Prospective registration
Public title	The effect of hydration and water supplementation on the cognitive performance of college students
Scientific title	Effect of Different Hydration State on Cognitive Performance
Approval of ethic committee	Peking University Institutional Review Committee
Ethical approval project identification code	IRB00001052-16071
Date of approved by ethic committee	5 December 2017
Study type	Interventional study
Study design	Randomized controlled trial
Key inclusion and exclusion criteria	Inclusion criteria: Aged between 18 and 23; Male and Female; In health state, without metabolic disease, oral diseases, and so on. Exclusion criteria: Aged < 18, or age > 23; With metabolic disease, oral diseases, and other diseases.
Interventions	Water supplementation
Outcomes	Urine osmolality, Blood pressure, Mood, Thirsty, Cognitive performances, MRI scans
Collecting samples	Urine, Blood
Recruitment state	Started
Randomization Procedure (please state who generates the random number sequence and by what method)	Primary sponsor generates random sequence with random number table

**Table 2:** The schedule of enrolment, interventions, and assessments.

Timepoint	Enrolment	Allocation	Post-Allocation	Close-Out
	5 April 2018	10 April 2018	15 April 2018	30 May 2018
Enrollment:				
Eligibility screen	X			
Informed consent	X			
Allocation		X		
Interventions:				
Water supplementation			X	
Assessments:				
Baseline anthropometric measurements variables: height, weight, waist circumference, blood pressure, blood glucose, body composition	X		X	
Outcome variables: cognitive performances, mood, subjective sensation, MRI			X	X

## Abbreviations

MRI	Magnetic Resonance Imaging
BOLD response	blood-oxygen-level-dependent response
WS group	Water supplementation group
NW group	No water supplementation group
H	Height
W	Weight
WC	Waist circumference
BP	Blood pressure
BG	Blood glucose
BC	Body composition
CP	Cognitive performance
VAS	Visual analogue
POMS	Profile of mood state

## References

[1] Jequier E., Constant F. (2010). Water as an essential nutrient: The physiological basis of hydration. Eur. J. Clin. Nutr..

[2] Savoie F.A., Kenefick R.W., Ely B.R., Cheuvront S.N., Goulet E.D. (2015). Effect of Hypohydration on Muscle Endurance, Strength, Anaerobic Power and Capacity and Vertical Jumping Ability: A Meta-Analysis. Sports Med..

[3] Popkin B.M., D’Anci K.E., Rosenberg I.H. (2010). Water, hydration, and health. Nutr. Rev..

[4] Pivarnik J.M., Palmer R.A. (1994). Water and electrolyte balance during rest and exercise. Nutrition in Exercise and Sport.

[5] Cian C., Barraud P.A., Melin B., Raphel C. (2001). Effects of fluid ingestion on cognitive function after heat stress or exercise-induced dehydration. Int. J. Psychophysiol..

[6] D’Anci K.E., Vibhakar A., Kanter J.H., Mahoney C.R., Taylor H.A. (2009). Voluntary dehydration and cognitive performance in trained college athletes. Percept. Mot. Skills.

[7] Ganio M.S., Armstrong L.E., Casa D.J., McDermott B.P., Lee E.C., Yamamoto L.M., Marzano S., Lopez R.M., Jimenez L., Le Bellego L. (2011). Mild dehydration impairs cognitive performance and mood of men. Br. J. Nutr..

[8] Sharma V.M., Sridharan K., Pichan G., Panwar M.R. (1986). Influence of heat-stress induced dehydration on mental functions. Ergonomics.

[9] Ely B.R., Sollanek K.J., Cheuvront S.N., Lieberman H.R., Kenefick R.W. (2013). Hypohydration and acute thermal stress affect mood state but not cognition or dynamic postural balance. Eur. J. Appl. Physiol..

[10] Armstrong L.E., Ganio M.S., Casa D.J., Lee E.C., McDermott B.P., Klau J.F., Jimenez L., Le Bellego L., Chevillotte E., Lieberman H.R. (2012). Mild Dehydration Affects Mood in Healthy Young Women. J. Nutr..

[11] Lopez-Sanchez J.I., Hancock P.A. (2017). Thermal effects on cognition: A new quantitative synthesis. Int. J. Hyperth..

[12] Hogervorst E., Riedel W., Jeukendrup A., Jolles J. (1996). Cognitive performance after strenuous physical exercise. Percept. Mot. Skills.

[13] Shirreffs S.M., Merson S.J., Fraser S.M., Archer D.T. (2004). The effects of fluid restriction on hydration status and subjective feelings in man. Br. J. Nutr..

[14] Pross N., Demazieres A., Girard N., Barnouin R., Santoro F., Chevillotte E., Klein A., Le Bellego L. (2013). Influence of progressive fluid restriction on mood and physiological markers of dehydration in women. Br. J. Nutr..

[15] Szinnai G., Schachinger H., Arnaud M.J., Linder L., Keller U. (2005). Effect of water deprivation on cognitive-motor performance in healthy men and women. Am. J. Physiol. Regul. Integr. Comp. Physiol..

[16] Duning T., Kloska S., Steinstrater O., Kugel H., Heindel W., Knecht S. (2005). Dehydration confounds the assessment of brain atrophy. Neurology.

[17] Kempton M.J., Ettinger U., Schmechtig A., Winter E.M., Smith L., McMorris T., Wilkinson I.D., Williams S.C., Smith M.S. (2009). Effects of acute dehydration on brain morphology in healthy humans. Hum. Brain Mapp..

[18] Nakamura K., Brown R.A., Araujo D., Narayanan S., Arnold D.L. (2014). Correlation between brain volume change and T2 relaxation time induced by dehydration and rehydration: Implications for monitoring atrophy in clinical studies. NeuroImage Clin..

[19] Kempton M.J., Ettinger U., Foster R., Williams S.C., Calvert G.A., Hampshire A., Zelaya F.O., O’Gorman R.L., McMorris T., Owen A.M. (2011). Dehydration affects brain structure and function in healthy adolescents. Hum. Brain Mapp..

[20] Biller A., Reuter M., Patenaude B., Homola G.A., Breuer F., Bendszus M., Bartsch A.J. (2015). Responses of the Human Brain to Mild Dehydration and Rehydration Explored In Vivo by ^1^H-MR Imaging and Spectroscopy. Am. J. Neuroradiol..

[21] Edmonds C.J., Crosbie L., Fatima F., Hussain M., Jacob N., Gardner M. (2017). Dose-response effects of water supplementation on cognitive performance and mood in children and adults. Appetite.

[22] Zhang N., Du S., Tang Z., Zheng M., Ma G. (2017). Effect of Water Supplementation on Cognitive Performances and Mood among Male College Students in Cangzhou, China: Study Protocol of a Randomized Controlled Trial. Int. J. Environ. Res. Publ. Health.

[23] Hu Y., Wu X., Hu Z., Ren A., Wei X., Wang X., Wang Y. (1999). Study of formula for calculating body surface areas of the Chinese adults. Acta Physiol. Sin..

[24] Bar-David Y., Urkin J., Kozminsky E. (2005). The effect of voluntary dehydration on cognitive functions of elementary school children. Acta Paediatr..

[25] Perrier E.T., Buendia-Jimenez I., Vecchio M., Armstrong L.E., Tack I., Klein A. (2015). Twenty-four-hour urine osmolality as a physiological index of adequate water intake. Dis. Markers.

[26] Hill A.J., Blundell J.E. (1982). Nutrients and behaviour: Research strategies for the investigation of taste characteristics, food preferences, hunger sensations and eating patterns in man. J. Psychiatr. Res..

[27] Short M., Lack L., Wright H. (2010). Does subjective sleepiness predict objective sleep propensity?. Sleep.

[28] Rolls B.J., Wood R.J., Rolls E.T., Lind H., Lind W., Ledingham J.G. (1980). Thirst following water deprivation in humans. Am. J. Physiol..

[29] Mcnair D.M. (1984). Citation Classic-Manual for the profile of mood states. Curr. Contents/Soc. Behav. Sci..

[30] Lichtenberger E.O., Kaufman A.S. (2012). Essentials of WAIS-IV Assessment.

[31] Conway A.R.A., Kane M.J., Bunting M.F., Hambrick D.Z., Wilhelm O., Engle R.W. (2005). Working memory span tasks: A methodological review and user’s guide. Psychon. Bull. Rev..

[32] Ekstrom R.B.R., French J.J.W., Harman H.H. (1976). Manual for Kit of Factor-Referenced Cognitive Tests.

[33] Xu S., Wu Z. (1986). The Construction of “The Clinical Memory Test”. Acta Psychol. Sin..

